# Multimodal cardiovascular magnetic resonance quantifies regional variation in vascular structure and function in patients with coronary artery disease: Relationships with coronary disease severity

**DOI:** 10.1186/1532-429X-13-61

**Published:** 2011-10-21

**Authors:** Ilias Kylintireas, Cheerag Shirodaria, Justin MS Lee, Colin Cunningon, Alistair Lindsay, Jane Francis, Matthew D Robson, Stefan Neubauer, Keith M Channon, Robin P Choudhury

**Affiliations:** 1 Department of Cardiovascular Medicine, University of Oxford and Oxford Centre for Clinical Magnetic Resonance Research (OCMR), Oxford, UK

**Keywords:** Atherosclerosis, magnetic resonance imaging, coronary artery disease

## Abstract

**Background:**

Cardiovascular magnetic resonance (CMR) of the vessel wall is highly reproducible and can evaluate both changes in plaque burden and composition. It can also measure aortic compliance and endothelial function in a single integrated examination. Previous studies have focused on patients with pre-identified carotid atheroma. We define these vascular parameters in patients presenting with coronary artery disease and test their relations to its extent and severity.

**Methods and Results:**

100 patients with CAD [single-vessel (16%); two-vessel (39%); and three-vessel (42%) non-obstructed coronary arteries (3%)] were studied. CAD severity and extent was expressed as modified Gensini score (mean modified score 12.38 ± 5.3). A majority of carotid plaque was located in the carotid bulb (CB). Atherosclerosis in this most diseased segment correlated modestly with the severity and extent of CAD, as expressed by the modified Gensini score (R = 0.251, P < 0.05). Using the AHA plaque classification, atheroma class also associated with CAD severity (rho = 0.26, P < 0.05). The distal descending aorta contained the greatest plaque, which correlated with the degree of CAD (R = 0.222; P < 0.05), but with no correlation with the proximal descending aorta, which was relatively spared (R = 0.106; P = n. s.). Aortic distensibility varied along its length with the ascending aorta the least distensible segment. Brachial artery FMD was inversely correlated with modified Gensini score (R = -0.278; P < 0.05). In multivariate analysis, distal descending aorta atheroma burden, distensibility of the ascending aorta, carotid atheroma class and FMD were independent predictors of modified Gensini score.

**Conclusions:**

Multimodal vascular CMR shows regional abnormalities of vascular structure and function that correlate modestly with the degree and extent of CAD.

## Background

Imaging biomarkers have proven useful in the evaluation of drugs used in the treatment of atherosclerosis [1,2]. A variety of invasive and non-invasive techniques have been applied to quantify plaque progression and regression,[3-9] including compositional [10], and metabolic changes [11]. The common goal is to extract reliable and reproducible quantitative data that yield mechanistic insights in small numbers of patients and in a short time frame.

Coronary artery atheroma burden can be estimated in partially stenosed arteries with intravascular ultrasound (IVUS) [8,12-14]. The principal drawback of IVUS is its invasive nature, although serious complications (such as arterial dissection or acute vessel closure) are relatively rare (< 0.5%), the use of IVUS is effectively limited to the study of patients in whom coronary angiography is clinically indicated. Because significant stenoses are generally treated with balloon angioplasty and stents, IVUS is further confined to interrogation of *non-stenotic *segments (< 50%) of a different, single coronary artery.

B-mode (2-dimensional) ultrasound can quantify thickening of the intima and media of carotid arteries (CIMT), with high spatial resolution. CIMT is safe, non-invasive, reproducible, quick and cheap to perform and can be standardized for application in multiple centres [15] and has been widely used in atherosclerosis treatment trials [16-22] However, while providing quantitative data on wall thickness, CIMT is limited to the carotid arteries and does not provide useful information on composition or function of the vessel wall.

Cardiovascular magnetic resonance (CMR) is emerging as a useful complementary modality in the assessment of response to therapy in atherosclerosis [4,10,23,24]. Compared to existing approaches, CMR offers several distinct advantages. Firstly, unlike CIMT, CMR is a volumetric technique that is not confined to single-plane imaging of the carotid arteries, but can be used to interrogate volumes of the carotid arteries bilaterally, the aorta and the peripheral arteries. Unlike IVUS, CMR is non-invasive, and does not require ionising radiation for catheter positioning. CMR is highly reproducible and capable of evaluating changes in plaque volume in relatively small numbers of patients [25-27]. Furthermore, CMR offers an opportunity to measure not only plaque burden but also plaque composition [10,28-30] and to provide physiological assessments of vascular function such as pulse wave velocity, aortic compliance and endothelial function in the forearm in a single integrated examination [24,31-33].

Previous studies did not take into account the distribution of atheroma along the studied vessels and did not quantitatively relate this to CAD extent or severity on a per segment basis [34,35]. As CMR is poised to become more widely applied for the evaluation of cardiovascular drug therapies, it has become important to define the relationship between MR derived indices of peripheral vascular structure and function and coronary disease.

Here, we determine (1) the extent and distribution of atheroma burden in the carotid arteries and aorta, (2) the degree and distribution of aortic distensibility, (3) carotid artery composition, and (4) flow mediated vasodilatation of the forearm, all measured by CMR, in patients with CAD and relate these to the findings obtained from clinically-driven coronary angiography. This cohort is likely to be representative of the general coronary artery disease population that comprises the bulk of patients who will eventually receive atherosclerosis-modifying drugs.

## Methods

### Study population

Patients admitted for coronary angiography for investigation of chest pain or abnormal non-invasive cardiac investigations and who had also participated in a vascular CMR research protocol were eligible for inclusion. This approach was designed to capture a cohort that was representative of the spectrum of coronary disease severity. All component studies were approved by the Local Research Ethics Committee. Written informed consent was obtained from all patients. Patients with known chronic inflammatory conditions, infection, malignancy, and contraindications to CMR scanning were excluded.

### Serum and plasma assays

Cholesterol and lipoprotein assays were performed using a Cobas-Mira Analyser (ABX Diagnostics, Shefford, UK). Total cholesterol was assayed using the enzymatic CHOD-PAP method and triglycerides were assayed using the enzymatic GPO-PAP method. HDL-cholesterol was assayed using a homogenous second generation PEGME method (Roche Diagnostics, Burgess Hill, UK).

### Coronary Angiography-Determination of the Severity of Coronary Atherosclerosis

X-Ray coronary arteriography was performed by the Judkins method. The severity (% stenosis) of coronary artery atherosclerosis was systematically estimated visually in each arterial segment, independently by two experienced observers, blinded to the clinical and CMR data. The coronary artery tree of each patient was scored using the modified Gensini method [36]. In this scoring system, a cumulative numeric score is determined by the degree of luminal narrowing and the anatomical location of each stenosis. The modified Gensini score has been described and validated previously [37]. The most severe stenosis in each of eight coronary segments was graded from 1 to 4 (1 = 1% to 49% lumen diameter reduction; 2 = 50% to 74% stenosis; 3 = 75% to 99% stenosis; 4 = 100% occlusion) to give a total score of between 0 and 32. This score therefore gives a measure that combines both the severity and extent of coronary atherosclerosis.

### CMR Scan-MR structural and functional vascular indices

CMR was performed on a 1.5 Tesla Sonata system (Siemens, Erlangen) at a constant temperature and after at least 20 minutes' quiet rest. Blood pressure was monitored using a sphygmomanometer.

### Aortic and carotid atheroma burden

**Figure 1:**
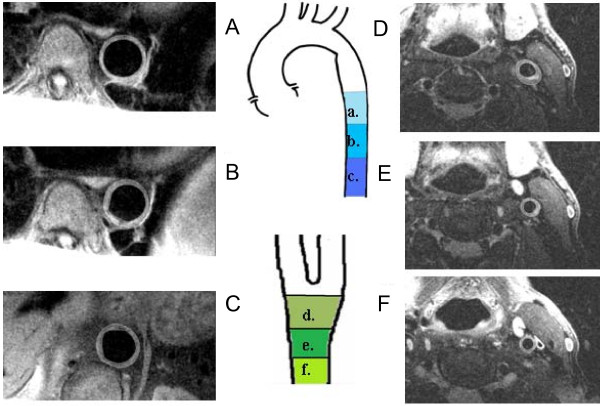
**(A-C) Carotid and (D-F) aortic segments**. (A-C) Carotid and (D-F) aortic segments. Characteristic turbo spin echo vessel wall images at the level of the proximal descending aorta (PDA) (a), middle descending aorta (MDA) (b), distal descending aorta (DDA) (c), carotid (bulb) (d), distal common carotid (PCC) (e) and proximal common carotid (DCC) (f).

For assessment of atherosclerosis in the aorta, a stack of 11 transverse black blood turbo spin echo (TSE) images covering the descending thoracic aorta was acquired during diastole (sequence parameters: TR 1 R-R interval, TE 11 ms, in-plane resolution 0.8 mm, slice thickness 5 mm). For assessment of atherosclerosis in the carotid arteries, we obtained 11 black blood turbo spin echo (TSE) cross-sectional images of both arteries, centred 1cm below the lowest point of the bifurcation (sequence parameters: FOV 150 mm, TR 2 R-R intervals, TE 81 ms, resolution 0.5 mm × 0.5 mm in plane, slice thickness 3 mm) (Figure 1).

All images were assessed using an image quality scoring system that was based on a previously published approach [38]. Images of quality score > 3 were analyzed. These images were segmented using semi-automated border detection algorithms developed using Matlab software (Mathworks Inc.) in order to define the inner (lumen) and outer vessel wall boundaries. Vessel wall area (WA) was calculated from the difference between these two contours, and also normalized to external vessel area to yield plaque index (PI), as previously described [24].

Mean carotid and aortic WA and PI were produced by averaging such measurements for the entire vessel (in the case of carotid indices the left and right vessels were combined to generate a mean value). To determine the distribution of atheroma along these vessels we produced segmental average WA and PI measurements for corresponding segments of the carotids [carotid bulb (CB), distal common carotid (DCC), and proximal common carotid (PCC)] and the aorta [proximal descending aorta (PDA), middle descending aorta (MDA) and distal descending aorta (DDA)].

### Carotid atheroma characterization

High resolution, black blood, proton density (TR/TE: 1200/12), T1 (TR/TE: 700/12), and T2 (TR/TE: 3200/81) weighted turbo spin echo (TSE) and bright blood steady state free precession imaging was used to characterize carotid plaque composition(in plane resolution 0.5 mm × 0.5 mm, slice thickness 3 mm for all types of images). Images of each carotid vessel (covering 6 mm proximally and 6 mm distally to the carotid bulb) were surveyed for atherosclerosis of the common carotid, carotid bulb, carotid bifurcation and internal carotid artery. For each subject the most severe plaque was identified and allocated a rank based on the previously published Modified American Heart Association (AHA) Atherosclerotic Plaque Classification for CMR (ranging from 1-8).

### Aortic distensibility

ECG-gated, steady state free precession (SSFP) 'cine' images were acquired during breath-hold to determine aortic distensibility. The first was obtained at the level of the right pulmonary artery through the ascending and proximal descending aorta and the second through the distal aorta below the diaphragm (CMR parameters: repetition time (TR) 2.8 ms, echo time (TE) 1.4 ms, in-plane resolution 2 mm, slice thickness 7 mm, temporal resolution 40 ms). Maximum and minimum aortic cross-sectional areas over the cardiac cycle were determined using semi-automated edge detection algorithms developed using Matlab software (Mathworks Inc.) (25). Distensibility was calculated as the relative change in area divided by the pulse pressure. Aortic distensibility was assessed at 3 levels of the thoracic aorta: ascending (AA), proximal descending (PA) and distal descending (DA) aorta. Semi automated software was used to track the vessel boundary during the cardiac cycle and calculate the relative cross-sectional area change in order to estimate aortic distensibility.

### Flow mediated dilatation

Steady state free precession (SSFP) acquisitions were used to determine brachial artery reactivity, as previously described [31]. SSFP Cross-sectional images of the brachial artery (typical parameters TR 6 ms, TE 3 ms, in-plane resolution 0.3 mm, slice thickness 3 mm) were acquired at baseline and following release of a cuff inflated to 50 mm Hg above systolic blood pressure on the forearm for five minutes. After a 10-minute interval, further brachial artery images were acquired following administration of 400 micrograms of sublingual glyceryl trinitrate (GTN). Post processing was performed using semi-automated edge detection methods developed with Matlab software (Mathworks Inc.). Maximum percentage change in brachial artery cross-sectional area at end-diastole was used to determine the response to each stimulus as previously described.

### Statistical analyses

Statistical analyses were carried out using SPSS version 15 (SPSS Inc.). Normal distribution of data was confirmed using the Kolmogorov-Smirnov test. Not Normally distributed data were logarithmically transformed for analysis. Normally distributed data are presented as mean ± standard error while not normally distributed data are presented as median (25^th^, 75^th ^percentile). One-way and repeated measures Analysis of Variance (ANOVA) with Bonferroni *post hoc *comparisons, Friedman's test, paired t tests and independent t test were used to compare numerical variables between groups as appropriate, while the chi-squared test was used for categorical variables. Pearson's coefficient was used for univariate correlation analysis. Stepwise multivariate regression analysis with backward elimination of independent variables was applied for the analysis of the association between CAD extent/severity and each of the MR derived vascular measures that had a significant univariate correlations with Gensini score. Independent variables were entered in the model if the level of significance of their correlation with the dependent variable was P < 0.05, while correlations were regarded as independent for variables retained in the model and reaching a P < 0.05 level of significance.

## Results

The study population comprised 100 patients who had undergone clinically-driven coronary angiography and who had participated in a vascular CMR research protocol (Table 1 for clinical characteristics). Accordingly, the population included patients with: single-vessel (16%); two-vessel (39%); and three-vessel (42%) coronary disease and non-obstructed coronary arteries (3%). The mean modified Gensini score was 12.38 ± 5.3 (range: 4 to 21).

**Table 1:** Clinical characteristics and risk factors.

Patient Characteristics	
Age	64.17 (± 0.78)
Gender	Male 88/100
	
Diabetes mellitus (1 or 2)	40/100
Hypertension	77/100
Smoking history	60/100
Hyperlipidemia	86/100
	
Total cholesterol	4.21 (± 0.09)
LDL	2.44 (± 0.09)
HDL	1.11 (± 0.03)
Triglycerides	1.87 (± 0.1)
	
Body mass index (BMI)	28.32 (± 0.41)
	
Aspirin	91/100
Clopidogrel	33/100
Statins	96/100
ACE inhibitors/ARBs	62/100
Beta blockers	86/100
Diuretics	26/100
Nitrates	13/100
Calcium channel blockers	28/100
Insulin	13/100
Oral hypoglycemic agents	26/100

### Carotid atheroma

In total, 2200 vessel MR images were obtained, of which 1520 were included in the analysis (69%). Vessels were excluded if fewer than 6 images met the inclusion threshold for analysis. This yielded 151 adequately imaged vessels (76 right and 77 left carotid arteries) out of the original 200. 83% of patients had at least 1 carotid artery amenable to analysis. The principal reason for exclusion was poor distinction between the outer vessel wall and the surrounding structures, rendering accurate wall area quantification impossible. The mean wall area was 29.8 mm^2 ^and the mean plaque index 0.42 (± 0.004). Mean wall area, but not mean plaque index, was slightly higher in the right compared to the left carotid artery [RCA wall area = 31.1 (25.5 - 35.8) mm^2 ^*vs*. LCA wall area = 29.6 (25.3 - 34.1; P < 0.05] [RCA plaque index = 0.41 (± 0.004) *vs*. LCA plaque index = 0.42 (± 0.005) mm^2 ^, not significant]. Inter-observer variability between the operators for wall area was < 4%. Intra-observer variability was < 1% on the images accepted for analysis.

Taken as a whole, there was no correlation between the mean wall area of the carotid arteries and the extent of coronary atheroma indicated by the modified Gensini score (R = 0.177, P = n.s.).

### Segmental distribution of carotid atheroma

**Figure 2:**
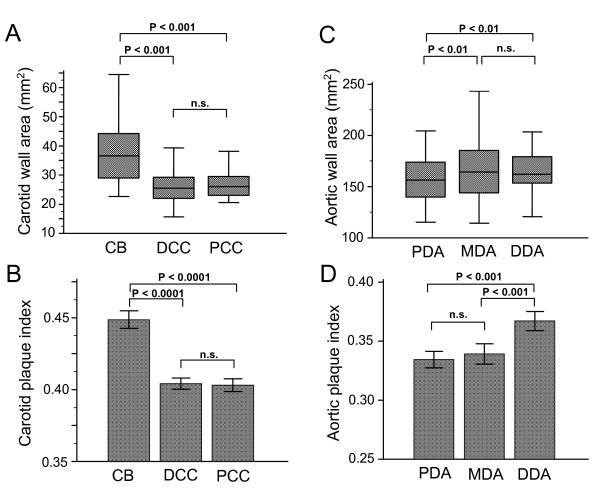
**Distribution of carotid and aortic plaque**. Distribution of carotid and aortic plaque. Atheroma burden (expressed as wall and plaque index) was higher in the carotid bulb (CB) compared to the distal (DCC) and proximal segments of common carotid (PCC) artery (A and B). Atheroma burden (expressed as wall area and plaque index) was higher in the distal (DDA) and middle descending (MDA) aorta in comparison to the proximal descending aorta (PDA) (C and D).

Examination of the segmental distribution of atheroma along the carotid artery revealed that the majority of carotid plaque was located in the carotid bulb (CB) (Figure 2A and 2B). Mean carotid bulb wall area correlated with the severity and extent of CAD, as expressed by the modified Gensini score (R = 0.251, P < 0.05). There was no association between the modified Gensini score and wall area of the proximal and distal common carotid.

### Carotid atheroma classification

**Figure 3:**
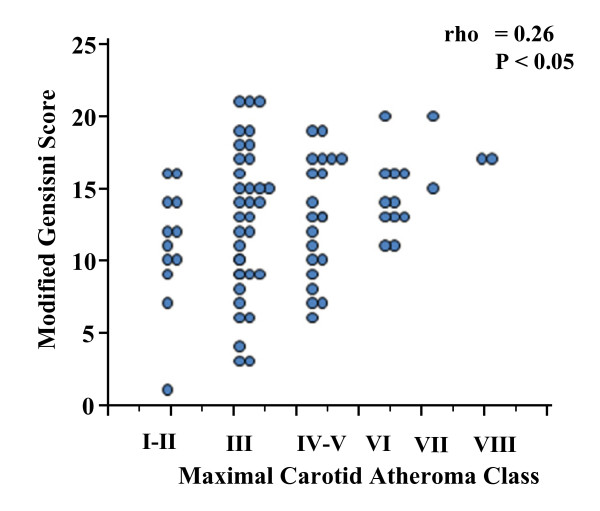
**Maximal carotid atheroma class *vs*. CAD severity**. Maximal carotid atheroma class *vs*. CAD severity. Maximal carotid atheroma class was positively associated with CAD severity.

On the basis of our image quality system we included in the analysis patients with adequate quality (vessel image score ≥ 3) visualization of the common carotid, bifurcation and internal carotid of at least one of their vessels. 80/100 patients had adequate imaging of at least one vessel and 75/100 had 2 assessable carotid arteries (Additional file 1: flow chart 1). Maximal carotid atheroma class was: I-II (normal and near-normal circumferential thickening) for 11/80 (13.75%), III (focal thickening) for 34/80 (42.5%), IV-V (lipid core) for 20/80 (25%), VI (plaque hemorrhage and fibrous cap rupture) for 10/80 (12.5%), VII (calcified plaque) for 3/80 (3.75%), VIII (fibrotic plaque) for 2/80 (2.5%). Maximal carotid atheroma class was associated with CAD severity (rho = 0.26, P < 0.05) (Figure 3).

### Aortic atheroma

Vessels of which < 6 images were analysable were excluded, the principal reason for exclusion being poor distinction between the outer vessel wall and the surrounding structures. In total, 807 out of 1100 (73%) aortic cross-sectional images were included in the analysis. This corresponded to 96 interpretable studies out of 100 obtained (Additional file 1: flow chart 1). The mean wall area was 168.3 (150.5 - 193.9) mm^2 ^and the mean plaque index was 0.3 (± 0.003). Inter-observer variability between the operators for wall area was < 3%. Intra-observer variability was < 1%. There was no significant correlation between mean aortic wall area and the severity of CAD (R = 0.198, P = n.s.).

### Segmental distribution of aortic atheroma

When aortic segments were considered the middle and distal descending aorta slices contained the greatest volume of plaque in comparison with the proximal descending aorta (see Figure 2C, &2D). As for the carotid arteries, a significant correlation emerged between CAD severity and wall area measured in the more diseased segments of aorta. For the middle descending aorta (R = 0.208; P < 0.05) and for the distal descending aorta (R = 0.222; P < 0.05), but with no correlation with the proximal descending aorta, which was relatively spared (R = 0.106; P = n. s.).

### Regional aortic distensibility analysis

**Figure 4:**
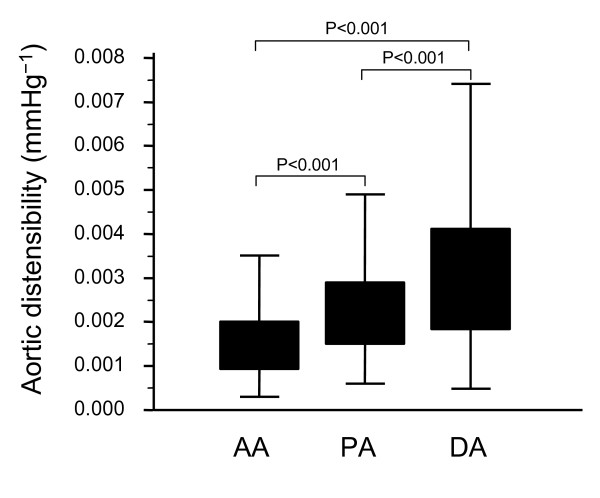
**Aortic distensibility along the thoracic aorta**. Aortic distensibility along the thoracic aorta. Of the three sites assessed [ascending (AA), proximal descending (PA) and distal descending (DA) aorta], distensibility was lowest at the AA and highest at the DA.

Aortic distensibility varied progressively along its length (ascending aorta: 1.8 ± 0.12 × 10^-3 ^mmHg^-1 ^, proximal descending aorta: 2.5 ± 0.14 × 10^-3 ^mmHg^-1 ^, distal descending aorta: 3.4 ± 0.21 × 10^-3 ^mmHg^-1 ^), with the ascending aorta being the least distensible segment (Figure 4).

### FMD vs. coronary atherosclerosis

**Figure 5:**
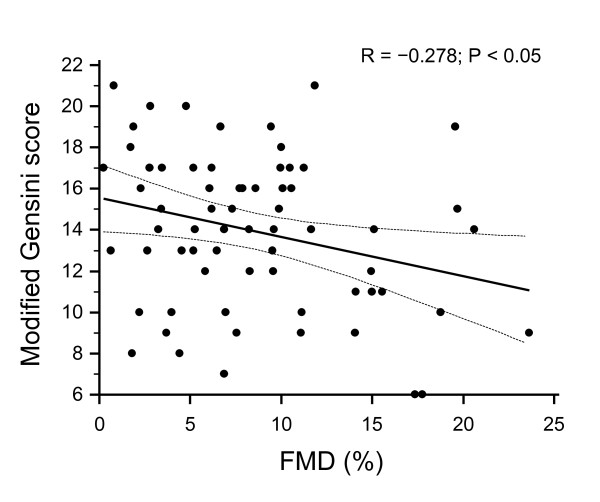
**Relationship of endothelial function, assessed by flow mediated vasodilatation of the brachial artery and extent of coronary artery disease**. Relationship of endothelial function, assessed by flow mediated vasodilatation of the brachial artery and extent of coronary artery disease. FMD inversely correlated with CAD extent and severity (as expressed by modified Gensini score).

Mean FMD in this population was 8.4 (0.7)%. Brachial artery FMD inversely correlated with modified Gensini score (R = -0.278; P < 0.05) indicating a quantitative relationship between the CAD severity and the degree of endothelial dysfunction (Figure 5).

### Multivariate analysis

Using multivariate regression analysis with inclusion of anthropometric (body mass index, waist hip ratio) and demographic data (age, gender), cardiovascular risk factors and MR indices as independent variables, as appropriate, none of the carotid plaque measurements and none of the classical risk factors emerged as independent predictors of modified Gensini score. Distal descending aorta atheroma burden, distensibility of the ascending aorta, maximal carotid atheroma class and FMD were the only independent predictors of modified Gensini score [β (SE) = 10.34 (0.08), P < 0.05, β (SE) = -7.29 (5.08), P < 0.005, β (SE) = 0.7(0.3), P < 0.05 and β (SE) = -0.19 (0.08), P < 0.05 respectively] (Table 2).

**Table 2:** Independent Predictors of Gensini score.

Predictors (R^2 ^= 0.17)	β (SE)	P
Distal descending aorta wall area	10.34 (0.08)	< 0.05
Ascending Aorta distensibility	-7.29 (5.08)	< 0.005
FMD	-0.19 (0.08)	< 0.05
Maximal Carotid Atheroma Class	0.7 (0.3)	< 0.05

Using multivariate regression analysis, Maximal Carotid Atheroma Class, distal descending aorta atheroma burden, distensibility of the ascending aorta and FMD were independent predictors of modified Gensini score.

## Discussion

We report the multimodal vascular CMR characteristics of 100 patients that represent a spectrum of coronary artery severity. In this clinically relevant cohort, we identify regional variations in both (i) the distribution of atherosclerosis in the aorta and carotid arteries and (ii) aortic distensibility, measured at three sites. For both carotid arteries and aorta, plaque burden of only the most diseased segments correlated modestly with the extent and severity of coronary disease. We also detect, for the first time, a relationship between increasing carotid atheroma complexity, characterized by CMR, and CAD extent/severity. The AHA atheroma classification based on histological features reflects in part the sequential development of atheromatous lesions [39]. On this basis, although plaques bearing features of vulnerability (i.e. IV-VI) are associated with higher risk of thromboembolism and cardiovascular events, higher class atheroma (i.e.VII-VIII) corresponds to more advanced atherosclerosis. The modest association that we detect between carotid atheroma classification and Gensini score is therefore pathophysiologically consistent with this theoretical scheme.

### Atherosclerosis imaging

According to conventional approaches to MR determination of atheroma burden, per patient measurements are derived by averaging or summation across the full extent of imaged aorta and/or the combined carotid vessels [4,9]. However, the current study demonstrates a propensity for MR-determined plaque in the carotid bulb and the practice of averaging along the vessel may diminish artificially the true range of plaque burden and thereby reduce the power for quantitative comparisons. The significance of the regional variation in distribution is further reinforced by the relationship with coronary atherosclerosis, estimated using a modified version of the Gensini scoring system. By virtue of being determined by both (i) the degree of coronary stenoses and (ii) the number of stenosed segments, this score provides an insight in the extent of coronary disease. Univariate analysis revealed that atheroma burden of the common carotid did not correlate with CAD severity, while only a modest correlation with atheroma of the carotid bulb was detected. This result is consistent with previous reports of a selective relationship between ultrasonographic atheroma measurements at that level and angiographic coronary disease estimates [40]. The correlation with angiographically-determined coronary disease was weak but comparable with that found in studies of ultrasound-measured IMT *vs*. quantitative coronary angiography [40]. No more than a modest correlation is to be expected, since CMR measures within-wall plaque volume, while coronary angiography estimates plaque extent based on the two-dimensional projection of lesions that encroach into the vessel lumen, with very limited ability to estimate the true size of plaques. Furthermore, patients included in this study had symptomatic angina; biasing inclusion to the presence of at least one flow-limiting coronary stenosis, irrespective of total coronary plaque burden. A recent study measuring intima media thickness along several segments of the carotid using ultrasound has not only confirmed significant differences in atheroma accumulation between segments but also demonstrated a selectively stronger relationship between coronary atherosclerosis and carotid intima media measurements at the level of the carotid bulb/bifurcation [40]. The importance of focusing segments of artery with most abundant atherosclerosis is further emphasized by the recent observation that larger wall areas show the greatest propensity for change (both regression and progression) over time [41]. Furthermore, larger plaques may show greater compositional heterogeneity, presenting an opportunity to quantify both total plaque size and lipid rich core [10,42].

Previous CMR studies of carotid atherosclerosis have largely focused on patients selected for the presence of significant carotid disease [38,43]. Using such an approach, often accompanied by comparison of MR appearances with histological examination of explanted endarterectomy material, it has been possible to determine and validate the use of multi-contrast-CMR for the assessment of plaque size and composition. The current study extends this analysis to patients that are more representative of the general coronary artery disease population and defines their CMR-derived vascular characteristics.

### Physiological measures

Aortic distensibility, determined by CMR is diminished in smokers;[31] type 2 diabetes;[32] and obesity [44]. In the Multi-Ethnic Study of Atherosclerosis, aortic distensibility at a single level was associated with increasing cardiovascular risk factors [45]. Importantly, distensibility varies over a substantial 'dynamic range' and can show improvement in response to treatment in a short time frame [9,44]. The measurement of lumen cross-sectional area is readily obtained using automated, highly reproducible techniques [46]. Here, we show marked regional differences in distensibility in patients with coronary artery disease. The reasons for diminished distensibility may not be simply explained. We and others have found that in some patients reduced distensibility is related to increased wall thickness, however this relationship does not hold in patients with diabetes where distensibility may be reduced to due compositional changes in the vessel wall, for instance due to cross linking of structural proteins by advanced glycation end products [32,47,48]. However, as we are normalizing distensibility measured at all three aortic sites to the same peripherally measured pulse pressure, the observed differences may not be directly related to vascular wall structure differences but rather to central pulse pressure differences along the length of the aorta. When testing the association with CAD severity we found that the relationship was only modest and limited to the ascending aorta.

Finally, the demonstrated lower variability of MR based FMD measurement in comparison to the corresponding ultrasonographic technique may account for detection of a modest, yet significant and independent, relationship between CAD severity and endothelial dysfunction in this study [49].

### Utility

CMR confers the ability to obtain multiple complementary measures of vascular structure and function in an integrated examination. This ability will enable the characterization of patients with different stages of disease who are likely to manifest a spectrum of vascular structural and functional dysfunction and to respond differently to given interventions. There is potential benefit in knowing which vascular segments and vascular parameters assessable by MR should be prioritized for specific studies, especially as part of a time consuming protocol and relatively expensive technique. We suggest that these results can help define MR scanning priorities.

## Conclusion

In patients with angiographic coronary artery disease, vascular CMR of the carotid arteries and aorta show atherosclerosis that is mostly apparent in the carotid bulb and distal descending aorta. Only the extent of plaque in these most diseased segments modestly correlated with the extent and severity of coronary disease. There were modest, yet independent, associations between CAD extent/severity and increasing carotid atheroma complexity, endothelial dysfunction and ascending aorta stiffening, as characterized by CMR. As CMR assumes greater importance for assessment of vascular structure and function, the parameters reported here will better inform the design of clinical trials.

## Competing interests

The authors declare that they have no competing interests.

## Authors' contributions

IK, CS and JMLcarried out the patient recruitment. IK, CS, JML, CC and JFcarried out the data acquisition. IK and CSperformed the image analysis. IK, CS, JML, CC and ALcontributed to the image reproducibility and quality analysis. RCP, CS and IKdeveloped the study hypothesis and concept. RCP, IK, SN and KMCinterpreted the results. IKperformed the statistical analysis. IK and RCPdrafted the manuscript. MDRdeveloped the MRI protocol and the image analysis software employed in this study. RCP, SN and KCMoffered scientific supervision and oversight for this project.

## Supplementary Material

Additional file 1 **Flow chart 1- Vessel wall image quality**. Flow chart highlighting the image quality criteria and demonstrating the image selection process for analysis of plaque burden (of the aorta and the carotids) and plaque composition (of the carotid arteries). Click here for file

## References

[B1] Duivenvoorden R Nederveen AJ de Groot E Atherosclerosis imaging as a benchmark in the development of novel cardiovasular drugs Curr Opin Lipidol 2007 18 613 21 10.1097/MOL.0b013e3282f19608 17993805

[B2] Lindsay AC Choudhury RP Form to function: current and future roles for atherosclerosis imaging in drug development Nat Rev Drug Discov 2008 7 517 29 10.1038/nrd2588 18483481

[B3] Brown BG Zhao XQ Chait A Simvastatin and niacin, antioxidant vitamins, or the combination for the prevention of coronary disease N Engl J Med 2001 345 1583 92 10.1056/NEJMoa011090 11757504

[B4] Corti R Fuster V Fayad ZA Lipid Lowering by Simvastatin Induces Regression of Human Atherosclerotic Lesions: Two Years' Follow-Up by High-Resolution Noninvasive Magnetic Resonance Imaging Circulation 2002 106 2884 7 10.1161/01.CIR.0000041255.88750.F0 12460866

[B5] Nissen SE Tuzcu EM Schoenhagen P Effect of Intensive Compared With Moderate Lipid-Lowering Therapy on Progression of Coronary Atherosclerosis: A Randomized Controlled Trial JAMA 2004 291 1071 80 10.1001/jama.291.9.1071 14996776

[B6] Bots ML Visseren FL Evans GW Torcetrapib and carotid intima-media thickness in mixed dyslipidaemia (RADIANCE 2 study): a randomised, double-blind trial Lancet 2007 370 153 60 10.1016/S0140-6736(07)61088-5 17630038

[B7] Kastelein JJ van Leuven SI Burgess L Effect of torcetrapib on carotid atherosclerosis in familial hypercholesterolemia N Engl J Med 2007 356 1620 30 10.1056/NEJMoa071359 17387131

[B8] Nissen SE Tardif JC Nicholls SJ Effect of torcetrapib on the progression of coronary atherosclerosis N Engl J Med 2007 356 1304 16 10.1056/NEJMoa070635 17387129

[B9] Lee JM Wiesmann F Shirodaria C Early changes in arterial structure and function following statin initiation: quantification by magnetic resonance imaging Atherosclerosis 2008 197 951 8 10.1016/j.atherosclerosis.2007.09.001 17977546 PMC2292239

[B10] Underhill HR Yuan C Zhao XQ Effect of rosuvastatin therapy on carotid plaque morphology and composition in moderately hypercholesterolemic patients: a high-resolution magnetic resonance imaging trial Am Heart J 2008 155 584 e1 8 10.1016/j.ahj.2007.11.018 18294500

[B11] Tahara N Kai H Ishibashi M Simvastatin attenuates plaque inflammation: evaluation by fluorodeoxyglucose positron emission tomography J Am Coll Cardiol 2006 48 1825 31 10.1016/j.jacc.2006.03.069 17084257

[B12] Nicholls SJ Sipahi I Schoenhagen P Application of intravascular ultrasound in anti-atherosclerotic drug development Nat Rev Drug Discov 2006 5 485 92 10.1038/nrd2040 16699493

[B13] Nissen SE Nicholls SJ Sipahi I Effect of Very High-Intensity Statin Therapy on Regression of Coronary Atherosclerosis: The ASTEROID Trial JAMA 2006 295.13.jpc60002 10.1001/jama.295.13.jpc60002 16533939

[B14] Nissen SE Yock P Intravascular Ultrasound: Novel Pathophysiological Insights and Current Clinical Applications Circulation 2001 103 604 16 11157729 10.1161/01.cir.103.4.604

[B15] Bots ML Evans GW Riley WA Carotid intima-media thickness measurements in intervention studies: design options, progression rates, and sample size considerations: a point of view Stroke 2003 34 2985 94 10.1161/01.STR.0000102044.27905.B5 14615619

[B16] Davidson M Meyer PM Haffner S Increased High-Density Lipoprotein Cholesterol Predicts the Pioglitazone-Mediated Reduction of Carotid Intima-Media Thickness Progression in Patients With Type 2 Diabetes Mellitus Circulation 2008 117 2123 30 10.1161/CIRCULATIONAHA.107.746610 18413496

[B17] Ludwig M Stapff M Ribeiro A Comparison of the effects of losartan and atenolol on common carotid artery intima-media thickness in patients with hypertension: results of a 2-year, double-blind, randomized, controlled study Clin Ther 2002 24 1175 93 10.1016/S0149-2918(02)80028-5 12182261

[B18] Mack WJ Selzer RH Hodis HN One-year reduction and longitudinal analysis of carotid intima-media thickness associated with colestipol/niacin therapy Stroke 1993 24 1779 83 10.1161/01.STR.24.12.1779 8248954

[B19] Meuwese MC de Groot E Duivenvoorden R ACAT inhibition and progression of carotid atherosclerosis in patients with familial hypercholesterolemia: the CAPTIVATE randomized trial JAMA 2009 301 1131 9 10.1001/jama.301.11.1131 19293413

[B20] Migdalis IN Gerolimou B Kozanidou G Effect of gemfibrozil on early carotid atherosclerosis in diabetic patients with hyperlipidaemia Int Angiol 1997 16 258 61 9543224

[B21] Sidhu JS Kaposzta Z Markus HS Effect of rosiglitazone on common carotid intima-media thickness progression in coronary artery disease patients without diabetes mellitus Arterioscler Thromb Vasc Biol 2004 24 930 4 10.1161/01.ATV.0000124890.40436.77 15001452

[B22] Taylor AJ Sullenberger LE Lee HJ Arterial Biology for the Investigation of the Treatment Effects of Reducing Cholesterol (ARBITER) 2: a double-blind, placebo-controlled study of extended-release niacin on atherosclerosis progression in secondary prevention patients treated with statins Circulation 2004 110 3512 7 10.1161/01.CIR.0000148955.19792.8D 15537681

[B23] Corti R Fuster V Fayad ZA Effects of Aggressive Versus Conventional Lipid-Lowering Therapy by Simvastatin on Human Atherosclerotic Lesions: A Prospective, Randomized, Double-Blind Trial With High-Resolution Magnetic Resonance Imaging Journal of the American College of Cardiology 2005 46 106 10.1016/j.jacc.2005.03.054 15992643

[B24] Lee JM Wiesmann F Shirodaria C Early changes in arterial structure and function following statin initiation: Quantification by magnetic resonance imaging Atherosclerosis 2007 10.1016/j.atherosclerosis.2007.09.001 PMC2292239 17977546

[B25] Saam T Kerwin WS Chu B Sample size calculation for clinical trials using magnetic resonance imaging for the quantitative assessment of carotid atherosclerosis J Cardiovasc Magn Reson 2005 7 799 808 10.1080/10976640500287703 16353440

[B26] Yuan C MRI of atherosclerosis in clinical trials NMR in Biomedicine 2006 19 636 54 10.1002/nbm.1065 16986119

[B27] Duivenvoorden R de Groot E Elsen BM In vivo quantification of carotid artery wall dimensions: 3.0-Tesla MRI versus B-mode ultrasound imaging Circ Cardiovasc Imaging 2009 2 235 42 10.1161/CIRCIMAGING.108.788059 19808598

[B28] Zhao XQ Phan BA Chu B Testing the hypothesis of atherosclerotic plaque lipid depletion during lipid therapy by magnetic resonance imaging: study design of Carotid Plaque Composition Study Am Heart J 2007 154 239 46 10.1016/j.ahj.2007.04.035 17643572

[B29] Zhao XQ Yuan C Hatsukami TS Effects of prolonged intensive lipid-lowering therapy on the characteristics of carotid atherosclerotic plaques in vivo by MRI: a case-control study Arterioscler Thromb Vasc Biol 2001 21 1623 9 10.1161/hq1001.098463 11597936

[B30] Choudhury RP Fuster V Badimon JJ MRI and Characterization of Atherosclerotic Plaque: Emerging Applications and Molecular Imaging Arterioscler Thromb Vasc Biol 2002 22 1065 74 10.1161/01.ATV.0000019735.54479.2F 12117718

[B31] Wiesmann F Petersen SE Leeson PM Global impairment of brachial, carotid, and aortic vascular function in young smokers: direct quantification by high-resolution magnetic resonance imaging J Am Coll Cardiol 2004 44 2056 64 10.1016/j.jacc.2004.08.033 15542292

[B32] Lee JM Shirodaria C Jackson CE Multi-modal magnetic resonance imaging quantifies atherosclerosis and vascular dysfunction in patients with type 2 diabetes mellitus Diab Vasc Dis Res 2007 4 44 8 10.3132/dvdr.2007.005 17469043 PMC2243181

[B33] Choudhury RP Fuster V Fayad ZA Molecular, cellular and functional imaging of atherothrombosis Nat Rev Drug Discov 2004 3 913 25 10.1038/nrd1548 15520814

[B34] Underhill HR Yuan C Terry JG Differences in carotid arterial morphology and composition between individuals with and without obstructive coronary artery disease: a cardiovascular magnetic resonance study J Cardiovasc Magn Reson 2008 10 31 10.1186/1532-429X-10-31 18549502 PMC2440371

[B35] Mani V Muntner P Gidding SS Cardiovascular magnetic resonance parameters of atherosclerotic plaque burden improve discrimination of prior major adverse cardiovascular events J Cardiovasc Magn Reson 2009 11 10 10.1186/1532-429X-11-10 19393089 PMC2680849

[B36] Gensini GG A more meaningful scoring system for determining the severity of coronary heart disease Am J Cardiol 1983 51 606 10.1016/S0002-9149(83)80105-2 6823874

[B37] Sullivan DR Marwick TH Freedman SB A new method of scoring coronary angiograms to reflect extent of coronary atherosclerosis and improve correlation with major risk factors Am Heart J 1990 119 1262 7 10.1016/S0002-8703(05)80173-5 1972310

[B38] Yuan C Mitsumori LM Ferguson MS In vivo accuracy of multispectral magnetic resonance imaging for identifying lipid-rich necrotic cores and intraplaque hemorrhage in advanced human carotid plaques Circulation 2001 104 2051 6 10.1161/hc4201.097839 11673345

[B39] Virmani R Kolodgie FD Burke AP Lessons from sudden coronary death: a comprehensive morphological classification scheme for atherosclerotic lesions Arterioscler Thromb Vasc Biol 2000 20 1262 75 10.1161/01.ATV.20.5.1262 10807742

[B40] Amato M Montorsi P Ravani A Carotid intima-media thickness by B-mode ultrasound as surrogate of coronary atherosclerosis: correlation with quantitative coronary angiography and coronary intravascular ultrasound findings Eur Heart J 2007 28 2094 101 10.1093/eurheartj/ehm244 17597051

[B41] Lee JM Robson MD Yu L-M High Dose Modified-release Nicotinic Acid Reduces Carotid Atherosclerosis: a Randomized, Placebo-controlled Magnetic Resonance Study J Am Coll Cardiol 2008 54 1787 94 10.1016/j.jacc.2009.06.036 19874992

[B42] Wasserman BA Sharrett AR Lai S Risk factor associations with the presence of a lipid core in carotid plaque of asymptomatic individuals using high-resolution MRI: the multi-ethnic study of atherosclerosis (MESA) Stroke 2008 39 329 35 10.1161/STROKEAHA.107.498634 18174475

[B43] Yuan C Zhang Sx SX Polissar NL Identification of fibrous cap rupture with magnetic resonance imaging is highly associated with recent transient ischemic attack or stroke Circulation 2002 105 181 5 10.1161/hc0202.102121 11790698

[B44] Rider OJ Francis JM Ali MK Beneficial cardiovascular effects of bariatric surgical and dietary weight loss in obesity J Am Coll Cardiol 2009 54 718 26 10.1016/j.jacc.2009.02.086 19679250

[B45] Malayeri AA Natori S Bahrami H Relation of aortic wall thickness and distensibility to cardiovascular risk factors (from the Multi-Ethnic Study of Atherosclerosis [MESA]) Am J Cardiol 2008 102 491 6 10.1016/j.amjcard.2008.04.010 18678312 PMC2586608

[B46] Jackson CE Shirodaria CC Lee JM Reproducibility and accuracy of automated measurement for dynamic arterial lumen area by cardiovascular magnetic resonance Int J Cardiovasc Imaging 2009 10.1007/s10554-009-9495-5 19779977

[B47] Shapiro BP Owan TE Mohammed SF Advanced Glycation End Products Accumulate in Vascular Smooth Muscle and Modify Vascular but Not Ventricular Properties in Elderly Hypertensive Canines Circulation 2008 118 1002 10 10.1161/CIRCULATIONAHA.108.777326 18711013 PMC2753480

[B48] Lin R-Y Choudhury RP Cai W Dietary glycotoxins promote diabetic atherosclerosis in apolipoprotein E-deficient mice Atherosclerosis 2003 168 213 20 10.1016/S0021-9150(03)00050-9 12801603

[B49] Leeson CP Robinson M Francis JM Cardiovascular magnetic resonance imaging for non-invasive assessment of vascular function: validation against ultrasound J Cardiovasc Magn Reson 2006 8 381 7 10.1080/10976640500526993 16669182

