# Supplementary material for: Multimodal cardiovascular magnetic resonance quantifies regional variation in vascular structure and function in patients with coronary artery disease: Relationships with coronary disease severity
Source: J Cardiovasc Magn Reson. 2011 Oct 21;13(1):61. doi: 10.1186/1532-429X-13-61 (PMC3256113; doi:10.1186/1532-429X-13-61)
Supplement: Additional file 1 — Flow chart 1- Vessel wall image quality. Flow chart highlighting the image quality criteria and demonstrating the image selection process for analysis of plaque burden (of the aorta and the carotids) and plaque composition (of the carotid arteries). [file 1532-429X-13-61-S1.DOC]

**Supplemental Figure 1.**
